# Spironolactone Eyedrop Favors Restoration of Corneal Integrity after Wound Healing in the Rat

**DOI:** 10.3390/ph16101446

**Published:** 2023-10-12

**Authors:** Daniela Rodrigues-Braz, Linxin Zhu, Emmanuelle Gélizé, Jean-Pierre Clarin, Xavier Chatagnon, Youcef Benzine, Philippe Rampignon, Agathe Thouvenin, Jean-Louis Bourges, Francine Behar-Cohen, Min Zhao

**Affiliations:** 1 Centre de Recherche des Cordeliers, Inserm, Université Paris Cité, Sorbonne Université, 75006 Paris, France; daniela.rodrigues-braz@inserm.fr (D.R.-B.); linxin.zhu@sorbonne-universite.fr (L.Z.); emmanuelle.gelize@sorbonne-universite.fr (E.G.); jean-louis.bourges@aphp.fr (J.-L.B.); min.zhao@inserm.fr (M.Z.); 2 Excelvision, Fareva, 07100 Annonay, France; 3 Valdepharm, Fareva, 27100 Val-de-Reuil, France; youcef.benzine6@gmail.com (Y.B.);; 4 CNRS, Inserm, UTCBS, Université Paris Cité, 75006 Paris, France; agathe.thouvenin@aphp.fr; 5 Département Recherche et Développement Pharmaceutique, Agence Générale des Equipements et Produits de Santé (AGEPS), AP-HP, 75005 Paris, France; 6 Ophtalmopole, AP-HP, Cochin Hospital, 75014 Paris, France; 7 Hôpital Foch, Service D’ophtalmologie, 92150 Suresnes, France

**Keywords:** spironolactone, drug development, topical administration, eyedrop, corneal wound healing, ocular surface, cornea

## Abstract

Abnormal corneal wound healing can compromise corneal transparency and lead to visual impairment. Mineralocorticoid receptor antagonists (MRA) are promising candidates to promote corneal remodeling with anti-inflammatory properties and lack gluococorticoids-associated side effects. In this preclinical study, a new polymer-free hydroxypropyl-gamma-cyclodextrin-based eyedrop containing 0.1% spironolactone (SPL), a potent but non-water-soluble MRA, was investigated for its ocular surface tolerance and efficacy in a rat model of corneal wound healing. SPL eyedrops were stable for up to 9 months at 4 °C. The formulation was well-tolerated since no morphological changes or inflammatory reactions were observed in the rat cornea after multiple daily instillations over 7 days. SPL eyedrops accelerated rat corneal wound healing, reduced corneal edema and inflammation, enhanced epithelial integrity, and improved nerve regeneration, suggesting restoration of corneal homeostasis, while potassium canrenoate, an active and soluble metabolite of SPL, had no effect. SPL eyedrops could benefit patients with impaired corneal wound healing, including that secondary to glucocorticoid therapy. Repurposing known drugs with known excipients will expedite translation to the clinic.

## 1. Introduction

The cornea is a transparent lens that allows the proper transmission of light towards the retina thanks to its complex and controlled avascular structure. The cornea is composed of cells, nerves, and stem cells, organized in layers, and well-organized extracellular components that maintain its transparency and self-healing capacity. The corneal epithelium, composed of 5 to 7 layers of cells, is about 50 μm in thickness and provides a smooth and regular surface made of nonkeratinized stratified squamous epithelium. The corneal epithelium forms a mechanical, structural, and immune barrier. It undergoes constant renewal by limbal stem cells. The stroma, produced by keratocytes that mostly reside in the anterior stroma, accounts for about 80% of the corneal thickness and its transparency results from the specific organization of collagen fibers and of the extracellular matrix where dendritic cells ensure immune surveillance. The endothelium is a single layer of hexagonal highly metabolically active cells that lay on the Descemet’s membrane and act as a pump regulating water content to contribute to corneal transparency. The cornea is one of the most innervated tissues in the body. The sensitive nerves derive from the trigeminal nerve. However, autonomic sympathetic nerve fibers also contribute to the corneal homeostasis and immune defense. The thick and straight stromal nerve trunks extend to plexiform networks which progressively form the thin nerve fibers within the stroma up to the epithelium [1]. The corneal nerves and the epithelium work synergistically to nourish the cornea and provide rapid epithelial wound healing and an ‘ad integrum’ repair to maintain corneal transparency and prevent inflammation and neovascularization. Any imbalance in these orchestrated processes taking place between the corneal elements might result in non-healing wounds, corneal ulceration, inflammation, neovascularization, defective innervation, scarring, oedema, and loss of transparency [2].

The loss of corneal transparency is the third most common cause of reduced vision and blindness in various conditions and diseases. It is estimated that around 4 million people worldwide experience visually significant corneal opacities [3]. A number of diseases or conditions can lead to corneal blindness including mechanical, chemical or photic trauma, infection, and autoimmune, metabolic, and genetic causes. Contact lens wear [4] and refractive surgeries are also common causes of corneal lesions [5]. Chronic corneal ulcerations, often resulting from abnormal wound healing associated with dry eye syndrome, herpetic and diabetic neurotrophic keratopathies [6], and immune-mediated peripheral ulcerative keratitis [7], threaten vision and eventually lead to corneal blindness.

The most recent advances in treating abnormal corneal wound healing have been reviewed in an excellent paper by Mohan et al. [3]. The authors conclude that, despite tremendous progress in corneal surgery and the promising results of gene and cell therapies, new treatments are still needed. Although a number of drugs are evaluated in animal models, the translation to clinical practice is the primary hurdle that could be overcome by the repurposing of known drugs [3].

Glucocorticoids (GCs) are endogenous hormones that exert pleiotropic effects, contributing to the immune privilege of the cornea, the maintenance of corneal transparency, and the control of epithelium regeneration. Cortisol has been measured in the aqueous humor [8,9,10,11,12] where it is highly available since no cortisol binding globulin has been measured in this fluid [12]. It has been hypothesized that local metabolism of cortisol could take place at the ocular surface as cortisol could be produced by corneal epithelial cells [13]. In clinical practice, to control inappropriate corneal inflammation and relieve patients’ symptoms, topical GCs are part of most therapeutic regimens, even in infectious keratitis [14], although they might delay corneal epithelial wound healing [15]. However, the effects of GCs vary as a function of the dose, the duration and timing of use, and is dependent on their chemical formulation. In addition, the exact mechanisms of action of GCs during corneal wound healing are not fully understood.

GCs act through binding to the glucocorticoid receptor (GR) and to the mineralocorticoid receptor (MR), both expressed in the cornea [15,16]. In mineralocorticoid-sensitive tissues like the kidney and the colon, MR is co-expressed with the 11-beta hydroxysteroid dehydrogenase type II enzyme (11β-HSD2) that inactivates GCs, preventing illicit MR-mediated GC effects. However, in tissues with low 11β-HSD2 expression such as the skin and the cornea, MR can be activated GCs and can cause inflammation, oxidative stress, and fibrosis [17]. In the skin and in the cornea, it has been demonstrated that the GCs-induced delay in wound healing was mediated by the activation of MR [18,19]. In the skin, topical pharmacological MR blockade has been shown to reduce inflammation and to accelerate wound healing delayed by GC both in healthy [18] and diabetic mice [20].

In transgenic mice invalidated for MR in vascular endothelial cells, we showed a reduction in corneal neovascularization in a model of limbal deficiency, showing that MR could also contribute to the maintenance of corneal avascularity [21]. In a rabbit model of corneal wound healing delayed by dexamethasone, we previously showed spironolactone (SPL), a potent steroidal MR antagonist formulated in polymeric nanomicelles significantly accelerated corneal re-epithelialization, whilst a potassium canrenoate solution, a soluble active metabolite of SPL, showed no effect, demonstrating the crucial role of formulation in obtaining a therapeutic effect [19]. Altogether, these results indicate that MR antagonists could be good candidates to favor corneal wound healing.

Several models and animal species can be used to evaluate the potential of new treatments for corneal wound healing. The rat offers the advantage of a large panel of antibodies to evaluate cell infiltration, corneal structures, and oxidative damages, whilst the rabbit is more appropriate for pharmacokinetic studies. Depending on the surface and severity of the applied heal, particularly if the limbus is resected, the model will be more appropriate to measure neovascularization and fibrosis [22]. In the case of a central epithelium resection, the model will be more appropriate to study the epithelial and stromal recovery after a calibrated wound. Having already demonstrated the potential of spironolactone to inhibit corneal neovascularization by limbal resection, and the beneficial effect of spironolactone nanomicelles in dexamethasone-induced wound healing delay, we chose in this work to study in more depth the effect of spironolactone on the restoration of corneal epithelium after its debridement in the rat.

To speed the translation to the clinic, our aims were as follows: to develop a polymer-free eyedrop formulation of SPL, a known drug repurposed for ocular use; to test the safety of the eyedrop in rats; and, to demonstrate the efficacy of the eyedrop in a rat model of corneal epithelial wound healing.

## 2. Results

### 2.1. Eyedrop Formulations Showed Good Physicochemical Stability at 4 °C for 9 Months

**Figure 1:**
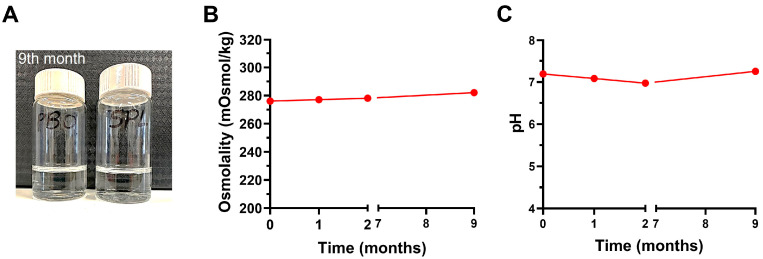
Stability of eyedrop formulations. (**A**) Spironolactone (SPL) and placebo (PBO) eyedrops remain clear and colorless after 9 months’ storage at 4 °C. (**B**) The osmolality of SPL eyedrops over time up to 9 months. (**C**) The pH of SPL eyedrops over time up to 9 months.

Both eyedrop formulations remained clear and colorless after 9 months’ storage in sterilized eyedroppers at 4 °C. No particle precipitation was observed (Figure 1A). The osmolality of SPL eyedrops did not vary significantly over time and was close to 280 mOsmol/kg when measured at 1 month, 2 months, and 9 months (Figure 1B). The pH of SPL eyedrops was 7.189 on day 0 and varied only slightly between 7.0 and 7.4 over a period of up to 9 months (Figure 1C).

### 2.2. SPL Eyedrops Showed No Sign of Corneal Integrity Disruption

To evaluate the tolerance of eyedrop formulations, both eyes of Lewis rats were treated with eyedrops containing 0.1% SPL or placebo (PBO) once or three times a day for 7 days. The corneal morphology and barrier integrity were evaluated by histology and immunofluorescence on day 8.

**Figure 2:**
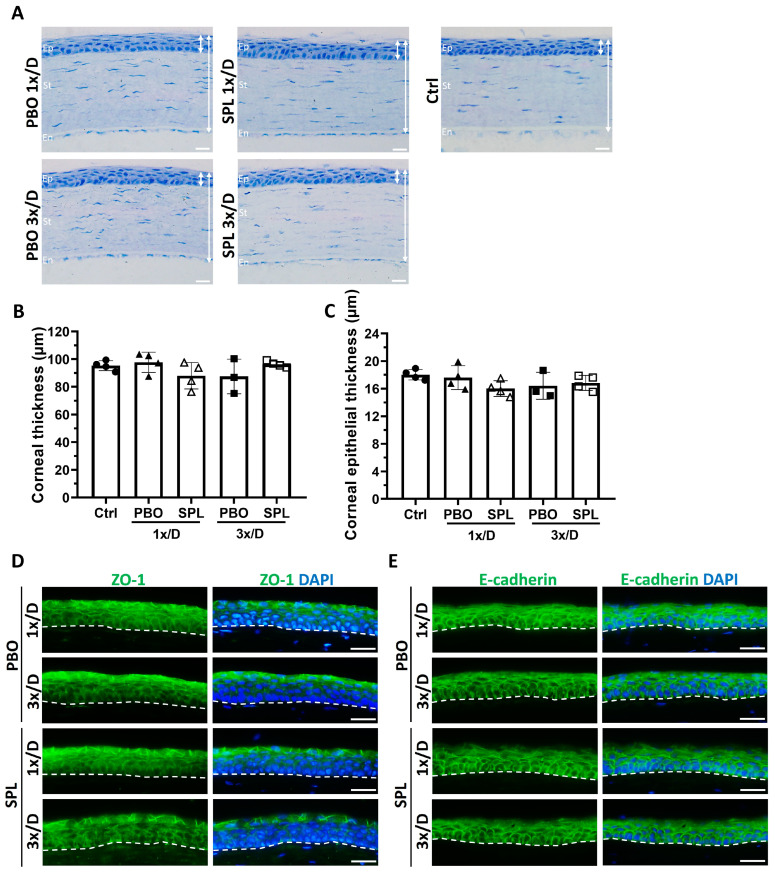
Rat corneal and corneal epithelial morphology after topical instillation of eyedrops containing spironolactone for 7 days. (**A**) Histological sections show normal corneal structure with stratified corneal epithelium (Ep), well-organized stroma (St), and intact corneal endothelial (En) monolayer after instillation of eyedrops containing 0.1% spironolactone (SPL) or placebo (PBO) once or three times a day for 7 days, similar to that of the untreated control cornea (Ctrl). Double-headed white arrows indicate the thickness of the cornea and corneal epithelium. Scale bars: 20 µm. (**B**) Quantification of the corneal thickness on histological sections shows no significant difference among all groups. Data are expressed as mean ± SD, n = 3–4 rat corneas. (**C**) Quantification of the corneal epithelial thickness shows no significant difference among all groups. Data are expressed as mean ± SD, n = 3–4 rat corneas. (**D**) ZO-1 immunofluorescence in green shows a cell membrane localization mainly in the superficial layers of the corneal epithelium. No disruption of the immunostaining was observed after topical treatment with SPL and PBO eyedrops. Dash lines indicate the inner limit of the corneal epithelium. Nuclei were counter-stained with DAPI in blue. Scale bars: 20 µm. n = 3–4 rat corneas. (**E**) E-cadherin immunofluorescence in green shows a cell membrane localization in all epithelial cells. No disruption of the immunostaining was observed after topical treatment with SPL or PBO eyedrops. Dash lines indicate the inner limit of the corneal epithelium. Nuclei were counter-stained with DAPI in blue. Scale bars: 20 µm. n = 3–4 rat corneas.

On histological sections, corneas treated with PBO or SPL formulations once or 3 times/day for 7 days presented a normal structure with stratified epithelium, regularly organized stroma, and a monolayer of endothelial cells, similar to untreated corneas (Figure 2A). The quantification of the total corneal thickness (Figure 2B) and corneal epithelial thickness (Figure 2C) did not show significant differences amongst all groups (*p* = 0.3762 and *p* = 0.2527 respectively). No corneal edema or inflammatory cell infiltration was observed after 7 days of treatment.

The corneal epithelial barrier was evaluated by the immunostaining of tight junction molecule ZO-1 and adhesion protein E-cadherin on day 8. ZO-1 staining was mainly located in the superficial layer of the corneal epithelium. We did not find any disruption of the staining in any treatment groups and no difference in localization was observed between PBO and SPL groups (Figure 2D). E-cadherin was expressed in the cell membrane of all epithelial cells, forming bridges between adjacent epithelial cells. There was no disruption or difference in its localization between PBO and SPL groups (Figure 2E). The inner border of the epithelium was regular, and epithelium layers were well defined by E-cadherin staining in all groups (Figure 2E).

### 2.3. SPL Eyedrops Did Not Induce Corneal Inflammation, Oxidative Stress, or Apoptotic Cell Death

**Figure 3:**
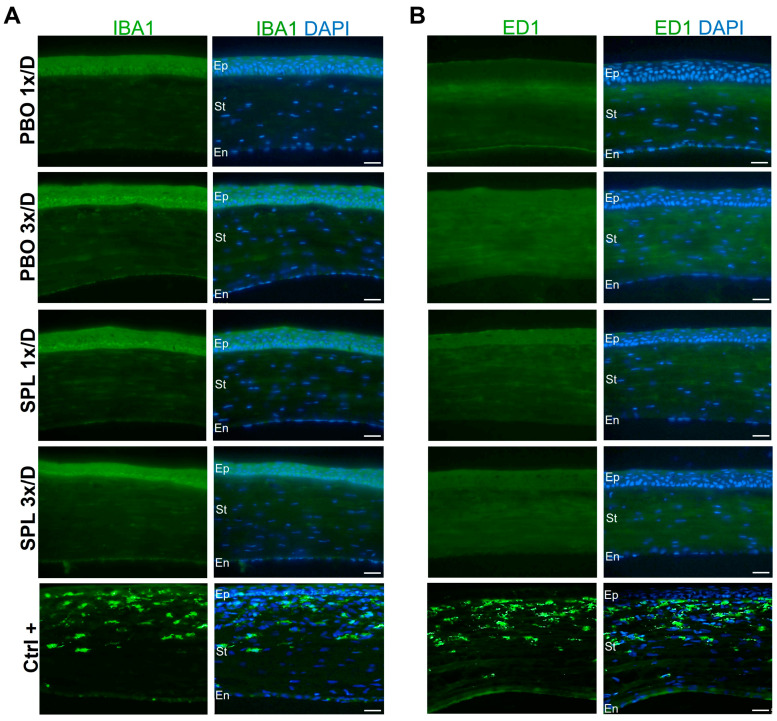
Immunostaining of inflammatory cells in rat corneas after topical instillation of eyedrops containing spironolactone for 7 days. (**A**,**B**) No IBA1- or ED1-positive inflammatory cells were observed in corneas treated with eyedrops containing 0.1% spironolactone (SPL) or placebo (PBO) once or 3 times a day for 7 days. Positive control corneas (Ctrl+) from an inflammation model show IBA and ED1 staining in green in inflamed corneas. Nuclei were counter-stained with DAPI in blue. Scale. Ep, epithelium; St, stroma; En, endothelium. Scale bars: 20 µm. n = 3–4 rat corneas.

**Figure 4:**
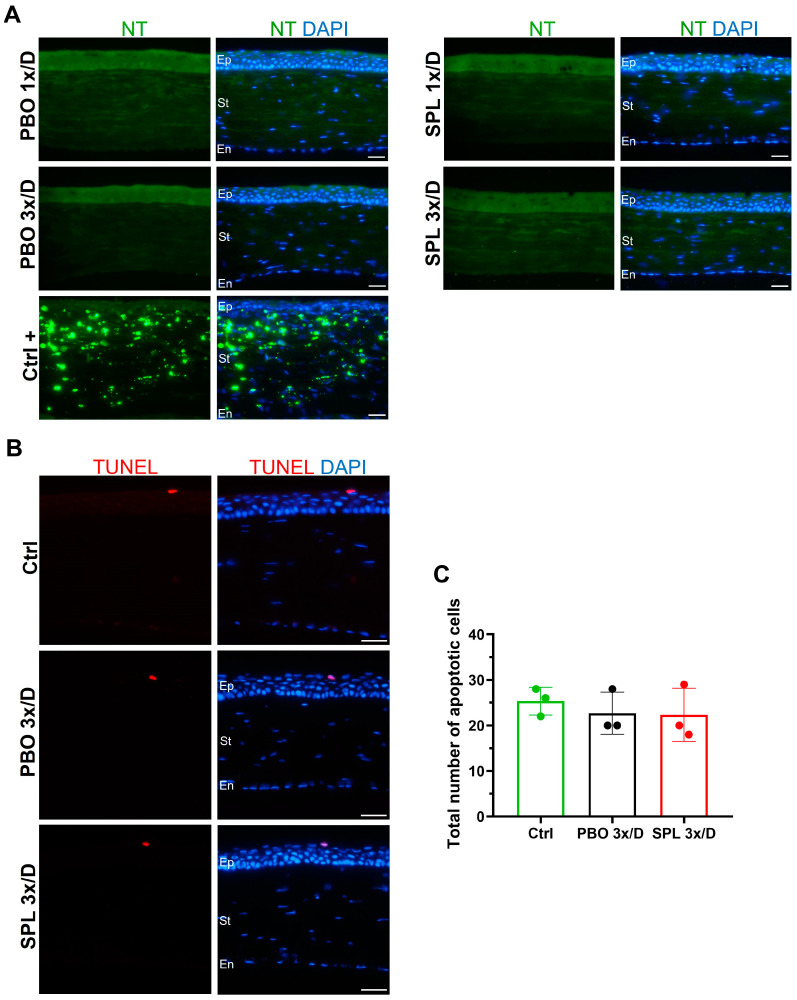
Nitrosative stress and cell death markers in rat corneas after topical instillation of eyedrops containing spironolactone for 7 days. (**A**) No nitrotyrosine (NT) positive cells were observed in corneas treated with eyedrops containing 0.1% spironolactone (SPL) or placebo (PBO) once or 3 times a day for 7 days. Positive control corneas (Ctrl+) from an inflammation model show NT staining in green. Nuclei were counter-stained with DAPI in blue. Ep, epithelium; St, stroma; En, endothelium. Scale bars: 20 µm. n = 3–4 rat corneas. (**B**) TUNEL positive apoptotic cells in red are only present in the superficial layers of the corneal epithelium in untreated control eyes (Ctrl) as well as in eyes treated with SPL or PBO eyedrops 3 times a day for 7 days. Nuclei were counter-stained with DAPI in blue. Scale bars: 20 µm. (**C**) Quantification of TUNEL positive cells across the whole corneal epithelium shows no significant differences amongst Ctrl, PBO, and SPL groups. Data are expressed as mean ± SD, n = 3 rat corneas.

IBA1 and ED1 immunostaining was performed on corneal cryosections to evaluate potential inflammatory cell infiltration. No IBA1- or ED1-positive inflammatory infiltrates were observed in the corneas on day 8 after PBO or SPL treatments (Figure 3). Nitrotyrosine (NT) is a marker of cell damage and nitrosative stress. There was no NT-positive immunostaining suggesting no nitrosative stress in any of the PBO and SPL groups (Figure 4A). TUNEL assay detects DNA fragmentation of apoptotic cells. It was performed on the corneas treated with eyedrops 3 times/day for 7 days. TUNEL-positive cells were located only in the superficial layer of the corneal epithelium in untreated normal corneas as well as in corneas treated with PBO or SPL (Figure 4B). The number of apoptotic cells across the whole epithelium was similar amongst groups (25.33 ± 3.06 in control, 22.67 ± 4.62 in PBO, and 22.33 ± 5.86 in SPL, *p* = 0.7214) (Figure 4C), suggesting that SPL eyedrops did not induce additional cell death.

Altogether, these results demonstrate the excellent tolerance of SPL eyedrops on the rat ocular surface.

### 2.4. SPL Eyedrops Improved Corneal Epithelial Healing and Reduced Corneal Edema in a Rat Model of Corneal Wound Healing

To investigate the therapeutic effects of SPL eyedrops, we used the rat model of corneal wound healing by mechanical injury, a model to study delayed corneal wound healing [23,24]. After central corneal de-epithelialization, both eyes were treated with 0.1% SPL eyedrops or PBO 3 times a day for 3 days. A group of rats was treated with 0.1% topical KCAN in PBS to assess the effects of the soluble active metabolite of SPL alone without eyedrop formulation.

**Figure 5:**
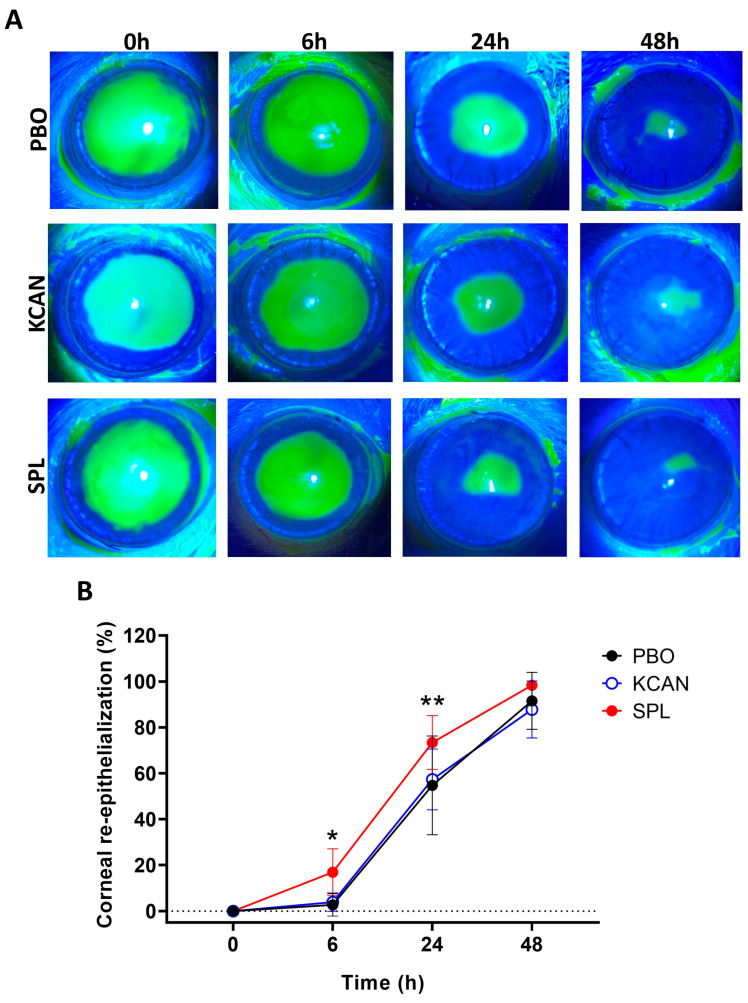
SPL eyedrops improve rat corneal re-epithelialization. (**A**) Fluorescein staining shows the 4-mm central corneal epithelial wound created at 0 h, and healing progress at 6, 24, and 48 h. Spironolactone (SPL) eyedrops improve corneal re-epithelialization compared to the placebo (PBO) and potassium canrenoate solution (KCAN). (**B**) Quantitative analysis shows that SPL eyedrops significantly increase the epithelial healing rate at 6 and 24 h compared with the PBO eyedrops and KCAN solution, while there is no difference between the KCAN and PBO groups. Data are expressed as mean ± SD, n = 7–9 rat corneas. *, *p* < 0.05; **, *p* < 0.01.

The healing progress was monitored with fluorescein staining to highlight the denuded area immediately (0 h), and 6, 24, and 48 h after corneal de-epithelialization as shown in Figure 5A. The healing rate of SPL-treated corneas was 16.91% ± 10.18 at 6 h, 73.41% ± 11.69 at 24 h, and 98.37% ± 2.25 at 48 h, while the healing rate of PBO eyedrops was 2.70% ± 4.89 at 6 h, 54.75% ± 21.49 at 24 h, and 91.56% ± 12.38 at 48 h, and that of KCAN solution was 3.89% ± 3.99 at 6 h, 57.33% ± 13.23 at 24 h, and 87.78%± 12.38 at 48 h. A significant improvement of corneal epithelial wound healing was observed in the SPL-treated group at 6 h as compared to the PBO- (*p* = 0.021) and KCAN- (*p* = 0.045) treated groups, and at 24 h as compared to the PBO- (*p* = 0.002) and KCAN- (*p* = 0.009) treated groups (Figure 5B), suggesting the superiority of the SPL eyedrop formulation over the PBO and unformulated KCAN solutions. No difference was observed between PBO and topical KCAN (Figure 5B) at 6 h (*p* = 0.970) and 24 h (*p* = 0.865). Corneal epithelial wounds closed on day 3 in all groups.

**Figure 6:**
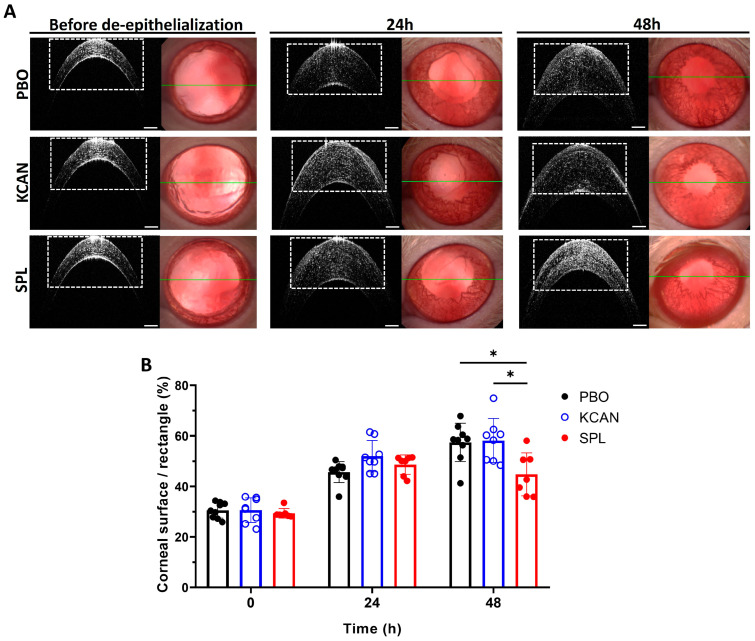
SPL eyedrops reduce rat corneal edema after corneal de-epithelialization. (**A**) In vivo B-scan optical coherence tomography shows the cross-sectional images (left) of the central cornea through the green line (right) before and 24 and 48 h after corneal de-epithelialization. Spironolactone (SPL) eyedrops reduce the corneal edema at 48 h as compared to placebo eyedrops (PBO) and potassium canrenoate solution (KCAN). The rectangles are of the same size and were used as a reference for quantitative analysis of corneal edema. (**B**) Corneal edema is presented as a percentage of the area of the reference rectangle occupied by the cornea. SPL eyedrops decrease corneal edema significantly compared to the PBO and KCAN groups at 48 h. Data are expressed as mean ± SD, n = 7–9 rat corneas. *, *p* < 0.05.

Corneal edema was assessed in vivo by OCT before and 24 and 48 h after corneal de-epithelialization, and expressed as a percentage of the area of the reference rectangle in Figure 6A occupied by the central cornea. Before epithelial debridement, corneal thickness was comparable amongst all groups (Figure 6A,B). Corneal edema occurred after corneal de-epithelialization. In the SPL-treated group, the corneal surface occupied 48.68% ± 3.86 of the reference rectangle at 24 h and 44.85% ± 8.52 at 48 h, while in PBO-treated group, the relative percentage was 45.73% ± 4.13 at 24 h and 57.47% ± 7.57 at 48 h; in KCAN-treated group, it was 52.07% ± 6.23 at 24 h and 58.26% ± 8.67 at 48 h. SPL eyedrops significantly reduced corneal thickening at 48 h compared to the PBO (*p* = 0.0234) and topical KCAN (*p* = 0.254) treatments (Figure 6A,B). There was no difference between the KCAN and PBO groups at 24 h (*p* = 0.074) and at 48 h (*p* = 0.979) (Figure 6B).

### 2.5. SPL Eyedrops Restored Epithelial Integrity in the Rat Model of Corneal Wound Healing

**Figure 7:**
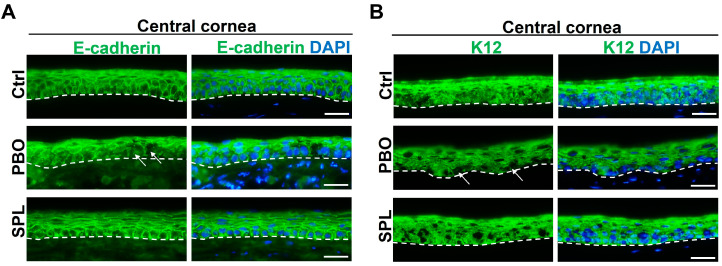
SPL eyedrops restore corneal epithelial integrity in rats after corneal wound healing. (**A**) Immunostaining of E-cadherin (green) shows discontinuity (arrows) and disorganization of E-cadherin in the epithelium of placebo-treated (PBO) corneas. Spironolactone eyedrops (SPL) restore E-cadherin continuity and the stratified structure of the corneal epithelium comparable to the control uninjured cornea (Ctrl). Dash lines indicate the inner border of the corneal epithelium. Nuclei were counter-stained with DAPI in blue. Scale bars: 20 µm. n = 4–5 rat corneas. (**B**) Immunostaining of cytokeratin 12 (K12, green) shows a decrease in K12 fluorescence and disruption (arrows) in the basal layer of the epithelium of PBO-treated corneas. The inner edge of the epithelium (dash line) is irregular. SPL eyedrops enhance K12 expression in the corneal epithelium and improve the regularity of the epithelial inner border, comparable to the control cornea. Nuclei were counter-stained with DAPI in blue. Scale bars: 20 µm. n = 4–5 rat corneas.

In order to evaluate the quality of the epithelial healing, we performed immunostaining of E-cadherin and cytokeratin K12, markers of differentiated corneal epithelium on corneal sections on day 5 after wound induction. Although the wound was healed in the PBO-treated corneas, there was a disorganization of E-cadherin and focal disruption in the basal layer of the corneal epithelium (Figure 7A). The structure of the epithelium was not well defined, and the inner border of corneal epithelium was irregular (Figure 7A). SPL eyedrops restored the continuity of E-cadherin and the stratified multilayer structure of the corneal epithelium, comparable to the control uninjured cornea (Figure 7A). Epithelial inner borders were more regular than in PBO-treated corneas.

In normal rat corneas, corneal epithelium expressed K12 with more intense expression in the superficial layers (Figure 7B). In PBO-treated corneas, we observed a general decrease in K12 expression and focal disruption in the basal layer of the epithelium. The inner border of the epithelium was irregular (Figure 7B). Topical SPL eyedrops enhanced the K12 expression and restored the structure of corneal epithelium (Figure 7B).

### 2.6. SPL Eyedrops Reduced Corneal Inflammatory Cell Infiltration in the Rat Model of Corneal Wound Healing

**Figure 8:**
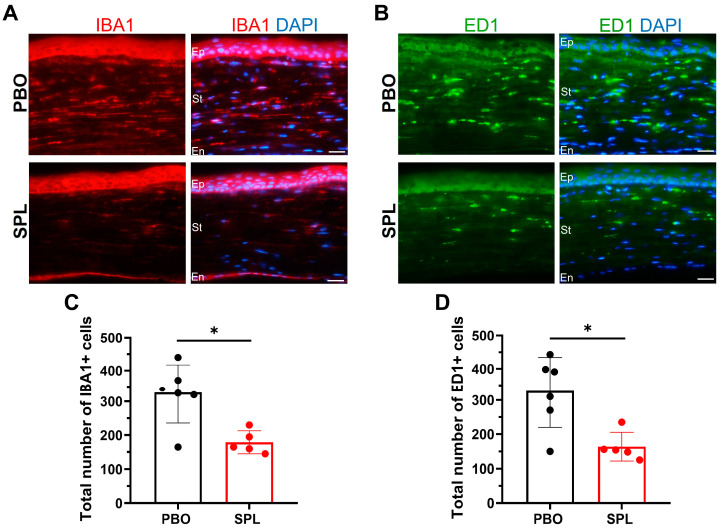
SPL eyedrops reduce inflammatory cell infiltration in rat corneas after corneal de-epithelialization. (**A**,**B**) IBA1 (in red) and ED1 immunostaining (in green) shows infiltration of inflammatory cells throughout the stroma of placebo-treated (PBO) corneas, whereas spironolactone eyedrops (SPL) limit the cell infiltration in the anterior stroma. Nuclei were counter-stained with DAPI in blue. Dash lines indicate the inner border of the corneal epithelium. Ep, epithelium; St, stroma; En, endothelium. Scale bars: 20 µm. (**C**,**D**) SPL eyedrops significantly decrease the number of IBA1- and ED1-positive cells in rat corneas compared with the PBO-treated group. Data are expressed as mean ± SD, n = 5–6 rat corneas. *, *p* < 0.05.

IBA1 and ED1 immunostaining were performed to evaluate the anti-inflammatory effect of SPL eyedrops. In PBO-treated group, numerous IBA1- and ED1-positive cells were detected throughout the corneal stroma, while SPL eyedrops limited the infiltration in the anterior stroma (Figure 8A,B). The number of IBA1-positive cells was 327 ± 91 in the PBO-treated group vs. 179 ± 34 in the SPL-treated group. The number of ED1-positive cells was 327 ± 107 in the PBO-treated group vs. 164 ± 42 in the SPL-treated group. SPL eyedrops significantly reduced the number of inflammatory cells in rat corneas (*p* = 0.0195 for IBA1 and *p* = 0.0303 for ED1) (Figure 8C,D).

### 2.7. SPL Eyedrops Improved Corneal Reinnervation in the Rat Model of Corneal Wound Healing

**Figure 9:**
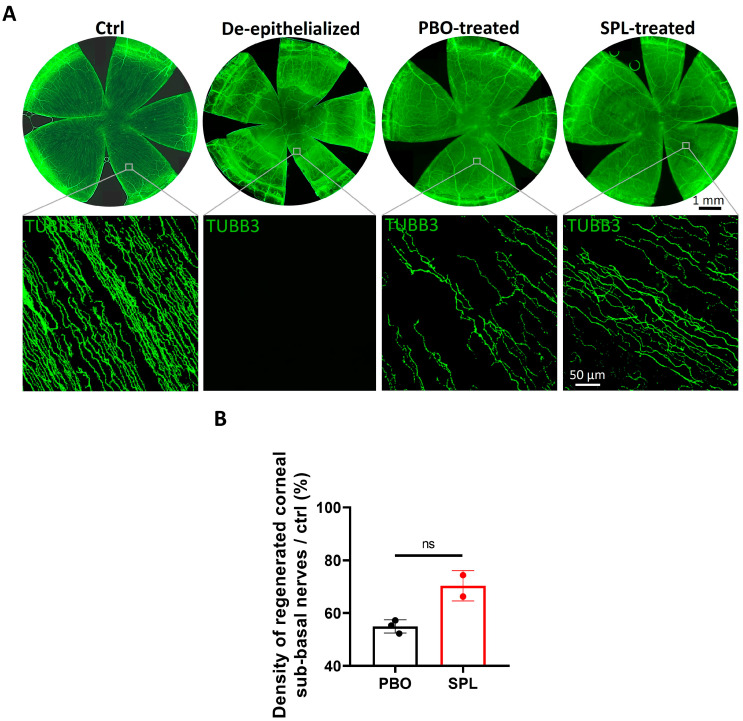
SPL eyedrops favor corneal re-innervation in the de-epithelialized central cornea in rats. (**A**) TUBB3 immunostaining in green on corneal whole mounts shows dense and linear corneal sub-basal nerves in control uninjured corneas (Ctrl). Corneal de-epithelialization mechanically removed the sub-basal nerve plexus. Spironolactone (SPL) eyedrops improve the morphology of regenerated sub-basal nerves compared with placebo-treated (PBO) corneas. (**B**) The density of regenerated sub-basal nerves is 54.95% relative to control cornea in the PBO-treated group (n = 3) and increased to 70.32% in the SPL-treated group (n = 2). Data are expressed as mean ± SD; ns, not significant.

We evaluated corneal reinnervation 5 days after topical PBO and SPL eyedrop treatments. In control uninjured corneas, TUBB3 immunostaining showed a dense linear architecture of the sub-basal nerves on corneal flat mounts. Corneal de-epithelialization mechanically removed the sub-basal nerve network (Figure 9A). Slow sub-basal nerve regeneration from the peripheral cornea to the de-epithelialized central cornea was observed in both PBO- and SPL-treated groups up to day 5 (Figure 9A). SPL eyedrops seemed to increase the sub-basal nerve density in the reinnervated corneal area compared to PBO-treated corneas. Moreover, the regenerated nerves appeared longer and more parallelly oriented in SPL-treated corneas than in PBO-treated corneas (Figure 9A). The density of corneal sub-basal nerve plexus was 54.95% ± 2.51 relative to control uninjured corneas in the PBO-treated group, and 70.32% ± 5.75 in the SPL-treated group, although the difference was not significant owing to the small sample size.

## 3. Discussion

Amongst several available MR antagonists, we chose SPL for the development of an eyedrop because SPL and its two main metabolites, 7α-thiomethylspironolactone and canrenone, are very potent MR antagonists with stable physicochemical properties and known systemic effects. The repurposing of old drugs offers the potential of quicker translation to the clinic; however, SPL is a steroid with very low water solubility (0.02 mg/mL) and a short half-life in ocular media (less than 1 h) [25], requiring proper formulation to increase its aqueous solubility and improve its corneal bioavailability. We and others have evaluated biodegradable polymers to enhance ocular drug bioavailability. For example, Poly(lactic-co-glycolic acid) (PLGA) has been used for the preparation of SPL microspheres to slowly release SPL for the treatment of diseases affecting the posterior segment of the eye [25]. The self-assembling Methoxy-poly(ethylene glycol)-di-hexyl-substituted-poly(lactic acid) (mPEG-dihexPLA) has been used to fabricate topical SPL micelles to treat corneal surface diseases [19]. Although they all have good safety profiles, the preparation of polymer-based formulations involves the use of organic solvents (e.g., ethanol, acetone) which can be harmful and might expose manufacturing limitations. The development of novel polymers necessitates extensive safety studies at a very high cost. Finally, empty polymer, the degradation products of which can lower the pH locally, might cause irritation and inflammation. For all these reasons, a polymer-free eyedrop formulation is preferred.

Cyclodextrins (CDs) are natural cyclic oligosaccharides; their molecules are donut-shaped with a hydrophilic outer surface and a hydrophobic inner cavity, making them well adapted to formulate hydrophobic drugs such as glucocorticoids [26,27]. CDs and their derivatives increase drug solubility and stability, enhance drug bioavailability, and reduce drug toxicity [26,27,28]. The US FDA lists 2-hydroxypropyl-β-cyclodextrin (HP-β-CD) and 2-hydroxypropyl-γ-CD (HP-γ-CD) as approved inert excipients. Examples of CDs-based eyedrops are indomethacin eyedrops (Indocollyre^TM^, Bausch & Lomb, Vaughan, ON, Canada) which contain HP-β-CD, diclofenac eyedrops (Voltaren Ophtha^TM^, Novartis, Basel, Switzerland) containing HP-γ-CD, and latanoprost which contains propylamino β-CD [28].

In this study, SPL was successfully dissolved in HP-γ-CD excipient at a concentration of 0.1%, a dose that proved effective in treating ocular surface defects in a rabbit model of GCs-induced wound healing delay [19]. The preliminary analysis showed encouraging stability of the SPL eyedrop formulation; however, further studies are required to measure SPL and its metabolites over time before it may be developed for clinical use. The bioavailability of SPL has not been evaluated by pharmacokinetic analysis in the present study since the rat model is not optimal for such analysis because of the small eye size and paucity of cells and tissues in the different corneal layers. A rabbit study will be conducted to provide a full quantitative evaluation of SPL and its metabolites in the different corneal layer over time. Nonetheless, we show herein that while potassium canrenoate, which is water soluble, is not efficient to speed up epithelial wound closure, the HP-γ-CD SPL formulation is efficient, which demonstrates that this formulation is essential to obtain the pharmacological effects of SPL.

In the healthy rat, the HP-γ-CD-based polymer-free SPL eyedrops showed an excellent tolerance as supported by the clinical, histological, and immunohistochemistry analysis, even following multiple topical instillations over one week. However, further studies are required to ensure that the clinical tolerability is good in humans.

In the rat model of corneal de-epithelialization, the 0.1% SPL eyedrops showed a significant beneficial effect on corneal wound healing by accelerating the speed of wound closure, decreasing corneal edema, improving epithelial integrity, and reducing inflammatory response. After corneal injury, the epithelium must heal in a timely manner to re-establish barrier function and regenerate the normal epithelial basement membrane so that the fibrotic stromal wound healing response can be terminated in favor of regenerative repair [5]. SPL eyedrops not only accelerate corneal re-epithelialization, but also improve epithelial cell adhesion and differentiation with cell layer stratification. Restoration of the epithelial integrity is important to prevent the entry of epithelial TGFβ and PDGF into the corneal stroma and avoid the transformation of keratocytes into corneal fibroblasts and myofibroblasts [29,30]. In addition, MR antagonists were shown to prevent TGF-β induced fibrosis in various models and organs such as atrial fibrosis [31], the kidney [32], the vascular endothelium [33], and the heart [34]. SPL could thus exert direct effects on keratocytes, and this should be explored.

SPL eyedrops also significantly reduced the infiltration of ED1 and IBA1 positive cells and their migration in the deep stroma. Whether the reduced cell infiltration results from the quicker restoration on the epithelial barrier or whether it is the primary effect of SPL cannot be determined; however, it confirms that SPL is released at an efficient concentration in the cornea as SPL did reduce IBA-1 positive cells in the rat model of laser-induced choroidal neovascularization [21] and in the diabetic retina [8]. Interestingly, in the glucocorticoid-impaired skin wound healing model, topical MR antagonists shorten the duration of inflammation by increasing the ratio of anti-inflammatory macrophages versus pro-inflammatory macrophages in wounds [35]. The role of activated and infiltrating dendritic cells in the healing process is complex and highly controlled since disrupted nerves can induce the activation of cells, which also interact with nerves to provide either protective or neurotoxic effects [36]. The exact role of SPL in these interaction remains to be explored but one major effector of the mineralocorticoid receptor, lipocalin 2, produced by dendritic cells, could be involved in this interaction [37].

Indeed, the integrity of nerve fibers is crucial for normal corneal function by sensing thermal, mechanical, and chemical stimuli leading to the release of essential neurotrophins for corneal homeostasis and wound healing [38]. Corneal diseases, such as diabetic and neurotrophic keratopathies, are associated with the impaired function of corneal nerves and epithelial breakdown resulting in delayed epithelial wound healing and corneal neurotrophic ulceration [6]. Unexpectedly, SPL eyedrops seems to help reinnervation of the cornea, which is essential for the return to homeostasis. The mechanism of action of SPL on the corneal nerve is under investigation. The upregulation of decorin by SPL, already demonstrated in the RPE/choroid, could be one of the mechanisms, as topical decorin, an important component of the extracellular matrix in the corneal stroma, was shown to favor the restoration of the sub-basal epithelial nerve plexus in a mouse model of benzalkonium chloride-induced corneal neuropathy together with a reduction of macrophage infiltration [39]. But mineralocorticoid antagonists could exert additional direct effects on corneal nerves since GR, MR, and 11β-HSD2 enzyme are expressed in Schwann cells [40] that play central and multifaceted roles in the maintenance and repair of peripheral nerves [41]. Further experiments are being conducted using transgenic rats overexpressing MR to decipher the exact role of MR in corneal neuroprotection.

We recognize several limitations, such as the fact that we did not use an analytical method to precisely quantify SPL and its metabolites over time; however, this was not within the scope of the study and there remains a need for further understanding of the neuroprotective mechanism of SPL. Other studies are ongoing, using other animal models such as transgenic rat overexpressing MR to this end.

## 4. Materials and Methods

### 4.1. Preparation of Eyedrop Formulations

Eyedrops containing 0.1% (*w*/*v*) SPL and placebo (PBO) eyedrops were respectively prepared according to the formula in Table 1. Pharmaceutical grade SPL was purchased from Azelis Pharma (Courbevoie, France) and HP-γ-CD was obtained from Wacker Chemie (Lyon, France). After dissolving HP-γ-CD in distilled water, SPL was added under stirring for at least 1 h at 20–25 °C until complete dissolution. Trometamol and NaCl were then added, and pH was measured and adjusted to 7.0–7.4 with 1N HCl. The osmolality of eyedrop formulations was around 280 mOsmol/kg. The PBO formulation was obtained using the same protocol without the addition of SPL.

### 4.2. Physicochemical Stability of Eyedrop Formulations

SPL and PBO eyedrops were stored in sterilized eyedroppers at 4 °C for 9 months. Their appearance was observed at 1, 3, 6, and 9 months. The pH and osmolarity were measured at 1 month, 3 months, and 9 months.

### 4.3. Animals

All experiments were performed in accordance with the European Communities Council Directive 8/609/EEC and French national regulations and approved by local ethical committees (#23478-2020010317557546 v4, Charles Darwin). Adult male Lewis rats (6–8 weeks, 200–250 g, Janvier, Le Genest-Saint-Isle, France) were used to assess the ocular tolerance of eyedrop formulations. Adult male Sprague-Dawley rats (12–13 weeks, 450–500 g, Janvier) were used to evaluate the therapeutic effects of SPL eyedrops. Animals were kept in pathogen-free conditions with *ad libitum* access to food and water and housed under a 12-h light/12-h dark cycle. Anesthesia was induced by intraperitoneal ketamine 100 mg/kg and xylazine 10 mg/kg. Rats were euthanized by Euthasol^®^ Vet 300 mg/kg.

### 4.4. Ocular Tolerance of Eyedrop Formulations

#### 4.4.1. Treatments

PBO or SPL (0.1%, *w*/*v*) eyedrops were instilled in both eyes of the rat. Animals were divided into four treatment groups: (1) SPL once a day for seven days; (2) PBO once a day for seven days; (3) SPL three times a day for seven days, and (4) PBO three times a day for seven days. At each time, two drops (~30 µL each) were administered with a 5-min interval between each administration. Untreated rats were used as controls. Rats were than euthanized on day 8, and eyes enucleated for histology and immunofluorescence.

#### 4.4.2. Corneal Histology

Eyes were fixed in 4% paraformaldehyde (PFA) and 0.5% glutaraldehyde for 2 h, dehydrated in a graded alcohol series, and embedded in historesin (Lecia, Heidelberg, Germany). Cross sections of 5 µm were obtained using a Leica Jung RM2055 Microtome and then stained with 1% toluidine blue. Corneal morphology was observed using a microscope (Olympus BX51, Rungis, France) equipped with a CCD camera (Olympus DP70) [8] For quantification, eye sections through the optic nerve head were photographed serially, from the limbus on one side of the cornea to the other. Corneal thickness and corneal epithelial thickness were measured every 200 µm using Fiji Image J software (ver. 1.54b; Wayne Rasband, National Institutes of Health, Bethesda, MD, USA). Twenty measurements were performed for each cornea; 3 to 4 corneas were used for each group.

#### 4.4.3. Immunofluorescence

Immediately after enucleation, eyes were snap-frozen in Tissue-Tek-OCT-compound (Bayer Diagnostics, Puteaux, France). Cryostat sections (10 µm) were fixed in 4% PFA, washed with PBS, and permeabilized with 0.1% Titron X-100 in PBS for 30 min. Unspecific binding sites were blocked with 5% normal goat serum for 30 min. Sections were then incubated with primary antibodies for 1 h at room temperature (RT), washed in PBS, and further incubated with secondary antibodies for 1 h at RT. After washing, slides were stained for 2 min with 4′,6-Diamidino-2-Phenyl-Indole (DAPI; 1:10,000), washed again with PBS, and mounted with gel mount (Dako, Agilent, Les Ulis, France) [8]. Negative control slides were stained without primary antibodies. Positive controls were corneal sections from rats with corneal inflammation. Images were taken using the Olympus fluorescence microscope. The following primary antibodies were used: rabbit anti-ZO-1 (1:200, Invitrogen ref. 40-2200, Waltham, MA, USA), mouse anti-E-cadherin (1:400, Abcam ref. ab231303, Cambridge, UK), rabbit anti-IBA1 (1:400, Wako ref. 019-19741, Richmond, VA, USA), mouse anti-ED1 (1:200, Bio-Rad ref. MCA341R, Colmar, France), and rabbit anti-Nitrotyrosine (1:200, Thermo Fisher Scientific ref. BS-8551, Saint Aubin, France). Secondary antibodies used were Alexa Fluor 488-conjugated goat anti-rabbit IgG (1:200; Thermo Fisher Scientific ref. A11008) and Alexa Fluor 488-conjugated donkey anti-mouse IgG (1:200; Thermo Fisher Scientific ref. A21202). Three to four corneas were used for each group.

#### 4.4.4. TUNEL Assay

TUNEL Assay was performed on eye cryosections according to manufacturer instructions (Roche Diagnostics, Mannheim, Germany). Nuclei were counter-stained with DAPI. TUNEL-positive cells were counted across the whole cornea on the sections at the level of the optic nerve head. Three corneas were used for each group.

### 4.5. Therapeutic Effects of SPL Eyedrop in a Rat Model of Corneal Wound Healing

#### 4.5.1. Corneal De-Epithelialization and Treatments

After general and local anesthesia (1% tetracaine, Sigma-Aldrich, Saint-Quentin-Fallavier, France), the central 4 mm of the cornea was marked with a 4-mm trephine. The corneal epithelium was then gently removed by a scarifier in the demarcated area without injuring the underlying corneal stroma. One of the following treatments was administered three times a day for three days on both corneas of rat: (1) eyedrops containing 0.1% SPL, (2) PBO eyedrops, or (3) PBS solution containing 0.1% potassium canrenoate (KCAN, Sigma-Aldrich), a water-soluble precursor of canrenone which is an active metabolite of SPL. Rat corneas without surface wounds and treatment were used as controls.

#### 4.5.2. In Vivo Optical Coherence Tomography and Slit Lamp Examination

Corneal morphology was assessed in vivo by optical coherence tomography (OCT, Micron III, Phoenix-Micron Inc., Bend, OR, USA) before and 24 and 48 h after corneal de-epithelialization. To measure the central corneal thickness, a rectangle was used as a reference on all corneal images. Results were expressed as a percentage of the area of the reference rectangle occupied by the cornea. Corneal epithelial defects were visualized by fluorescein staining under slit lamp immediately (0 h), and 6, 24, and 48 h after de-epithelialization. At each time point, the healing rate was calculated as a percentage reduction in the fluorescein-stained area compared to the wound created at 0 h. Images from seven to nine corneas were analyzed at each time point for each group.

#### 4.5.3. Immunofluorescence on Corneal Sections and on Whole-Mounted Corneas

Rats were euthanized 5 days after corneal de-epithelialization. Eyes were used for cryosection and corneal flat mounting. Immunofluorescence on corneal sections were performed as previously described. Primary antibodies used were mouse anti-E-cadherin, rabbit anti-Cytokeratin 12 (1:400, Abcam ref. ab185627, Cambridge, UK), rabbit anti-IBA1, and mouse anti-ED1. Secondary antibodies used were Alexa Fluor 488-conjugated goat anti-rabbit IgG, Alexa Fluor 488-conjugated donkey anti-mouse IgG, and Alexa Fluor 594-conjugated goat anti-rabbit IgG (1:200, Thermo Fisher Scientific ref. A11012). The number of IBA1- and ED1-positive inflammatory cells was counted for the entire corneal section. One section per cornea, and 5 to 6 corneas were analyzed for each group.

For corneal whole mounts, eyes were fixed in 1.3% PFA for 45 min at room temperature. After washing with PBS, corneas were excised along the sclerocorneal rim, cut into four to five petals, and blocked and permeabilized with PBS containing 5% normal goat serum and 0.5% Triton X-100 for 1 h. Corneas were then incubated with a mouse anti-Tubulin β III (TUBB3) antibody (1:500, Biolegend ref. 801202, San Diego, CA, USA) diluted in PBS containing 5% normal goat serum, 0.1% Triton X-100 and 0.1% Tween 20 (Sigma Aldrich, St. Louis, MO, USA) for 5 days at 4 °C. After washing, corneas were incubated with Alexa 488-conjugated donkey anti-mouse IgG (1:200, Thermo Fisher Scientific ref. A21202) overnight at 4 °C. Images of TUBB3 staining were observed and captured using a confocal microscope (Zeiss LSM 710 Oberkochen, Germany). For nerve quantification, ten z-stacks of the sub-basal nerves in the reinnervated corneal area were used. The density of corneal sub-basal nerves was measured using the Fiji Image J software, version 1.54b. The percentage of sub-basal nerve density in PBO- (n = 3) or SPL-treated corneas (n = 2) to that in normal control corneas was calculated.

### 4.6. Statistics

A study of the literature and an estimation using G-power software, version 3.1.9.6, were carried out to estimate the number of animals required. Data are expressed as mean ± SD. Statistical analysis was performed using the GraphPad Prism (GraphPad Software, version 8, San Diego, CA, USA). The Mann–Whitney test was used to compare two groups. The Kruskal–Wallis test followed by Dunn’s test was used to compare more than two groups. For grouped data, a two-way ANOVA was used followed by Tukey’s test. A *p* value less than 0.05 was considered statistically significant.

## 5. Conclusions

In conclusion, we have developed a polymer-free HP-γ-CD-based SPL eyedrop formulation for the treatment of corneal wounds that shows good preliminary stability and excellent tolerance over one week of repeated instillation. As compared to potassium canrenoate, which had no effect on corneal wound healing in the rat, the efficacy of SPL eyedrops to deliver efficient drug levels is attested to by its beneficial effects on the speed of corneal re-epithelialization, the reduction of corneal edema and inflammation, and the improvement of nerve regeneration. The SPL eyedrops could benefit patients with impaired corneal wound healing, including patients where this occurs secondary to GC use.

## Figures and Tables

**Table 1:** Composition of SPL and PBO eyedrops.

Compound	Spironolactone	Placebo
HP-γ-CD	3.00 g	3.00 g
Spironolactone	0.10 g	-
Trometamol	0.60 g	0.60 g
HCl (1 N, q.s)	pH = 7.2	pH = 7.2
NaCl	0.50 g	0.50 g
Distilled water (q.s)	100.00 mL	100.00 mL

## Data Availability

Data is contained within the article.
